# Chemerin as a biomarker of inflammatory bowel diseases: a meta-analysis

**DOI:** 10.1186/s12876-025-04132-2

**Published:** 2025-09-26

**Authors:** Zhi Tian, Qun Xiang, Meichun Long, Jiangtao Liao, Fang Wang, Xuefeng Li

**Affiliations:** 1 Department of Gastroenterology, People’s Hospital of Xiangxi Autonomous Prefecture / The First Affiliated Hospital of Jishou University / Clinical College of Jishou University, Jishou, 416000 China; 2 Department of Digestive Diseases, People’s Hospital of Hunan Province, Changsha, 410021 China

**Keywords:** Inflammatory bowel disease, Chemerin, Crohn’s disease, Ulcerative colitis, Meta-analysis

## Abstract

**Purpose:**

Chemerin, an adipokine involved in immune regulation and inflammation, has been implicated in the pathogenesis of inflammatory bowel disease (IBD), including Crohn’s disease (CD) and ulcerative colitis (UC). However, existing studies have reported inconsistent findings regarding its diagnostic value. This meta-analysis aimed to clarify the association between blood chemerin levels and IBD.

**Methods:**

A systematic search was conducted in PubMed, Embase, Web of Science, Wanfang, and China National Knowledge Infrastructure (CNKI) up to February 10, 2024. Observational studies comparing blood chemerin levels between IBD patients and healthy controls, or between active and non-active IBD, were included. Standardized mean differences (SMDs) with 95% confidence intervals (CIs) were pooled using a random-effects model by incorporating the possible influence of the heterogeneity.

**Results:**

Ten case-control studies with 18 datasets involving 799 CD patients, 520 UC patients, 609 healthy controls, were included. Compared with healthy controls, IBD patients had significantly higher blood chemerin levels (SMD: 0.61, 95% CI: 0.46–0.76, *p* < 0.001; I² = 48%). This association remained consistent across subgroups by disease type, study quality score, study region, and BMI matching (*p* for subgroup difference all > 0.05). Furthermore, chemerin levels were higher in active versus non-active IBD patients (SMD: 0.36, 95% CI: 0.15–0.57, *p* < 0.001; I² = 38%), with robust findings across subgroup and sensitivity analyses. No significant publication bias was detected.

**Conclusions:**

Elevated blood chemerin levels are associated with both the presence and activity of IBD, supporting its potential role as a non-invasive biomarker for disease diagnosis and monitoring.

**Supplementary Information:**

The online version contains supplementary material available at 10.1186/s12876-025-04132-2.

## Introduction

Inflammatory bowel disease (IBD) is a group of chronic, relapsing inflammatory disorders of the gastrointestinal tract, primarily comprising Crohn’s disease (CD) and ulcerative colitis (UC) [1, 2]. CD can affect any part of the gastrointestinal tract from mouth to anus and is characterized by transmural inflammation, while UC typically involves the colon and rectum with inflammation limited to the mucosal layer [3]. The global burden of IBD has risen substantially over the past decades, with increasing incidence and prevalence not only in Western countries but also in newly industrialized regions, including Asia [4, 5]. IBD is associated with significant morbidity, impaired quality of life, and increased risk of colorectal cancer [6]. Recent therapeutic advances, including targeted small-molecule agents such as Janus kinase (JAK) inhibitors, have expanded treatment options for moderate-to-severe IBD [7]. Although not directly life-threatening in most cases, the chronic nature of IBD, potential complications, and need for long-term treatment make early diagnosis and monitoring of disease activity essential for improving patient outcomes [8].

Chemerin is a multifunctional adipokine encoded by the RARRES2 gene [9]. It is secreted as an inactive precursor and becomes biologically active following proteolytic cleavage by inflammatory and coagulation-related proteases [10]. Chemerin plays a crucial role in immune modulation, adipogenesis, glucose homeostasis, and inflammation through its interaction with receptors such as CMKLR1, GPR1, and CCRL2 [11]. In the context of inflammation, chemerin functions as a chemoattractant for immune cells, particularly macrophages and dendritic cells, and is involved in the regulation of both innate and adaptive immune responses [11, 12]. Given its immunoregulatory properties, chemerin has been implicated in various chronic inflammatory diseases, including obesity-related metabolic disorders, cardiovascular disease, and autoimmune conditions [13–15].

Recent studies have suggested a potential link between chemerin and the pathogenesis of IBD [16, 17]. Chemerin may contribute to intestinal inflammation through several mechanisms, including modulation of leukocyte recruitment, regulation of cytokine production, and interaction with gut microbiota [17, 18]. Elevated levels of chemerin have been observed in patients with active IBD, and its expression has been detected in inflamed intestinal tissues [19, 20]. However, findings across studies have been inconsistent, with some reporting significantly higher circulating chemerin levels in IBD patients compared to healthy controls [21–27], while others have found no significant difference [28–30]. Moreover, no prior meta-analysis has comprehensively quantified the difference in chemerin levels between IBD patients and healthy controls, or between active and inactive disease states. This lack of pooled evidence limits our understanding of chemerin’s diagnostic or monitoring potential in clinical practice.

To date, although several studies have investigated the relationship between circulating chemerin levels and IBD, their findings remain inconsistent, and no prior meta-analysis has comprehensively synthesized these data [15–17]. Existing reviews on IBD biomarkers have primarily focused on conventional markers such as fecal calprotectin or C-reactive protein and have not quantitatively evaluated chemerin’s diagnostic or activity-monitoring value [31, 32]. Our meta-analysis is the first to systematically evaluate the association between blood chemerin levels and both the presence and activity of IBD, including CD and UC. By pooling data from recent observational studies and conducting rigorous subgroup and sensitivity analyses, this study aims to clarify chemerin’s role as a potential non-invasive biomarker for IBD diagnosis and monitoring, thereby addressing an important gap in the literature.

### Methods

In conducting this meta-analysis, the study adhered to the guidelines of PRISMA 2020 [33, 34] and the Cochrane Handbook for Systematic Reviews and Meta-analyses [35], including the protocols for study design, data extraction, statistical analysis, and results presentation. Additionally, the meta-analysis protocol was registered with the International Prospective Register of Systematic Reviews (PROSPERO) under registration identifier CRD420251004056. The detailed search strategy for each database is shown in Supplemental File 1.

### Literature search

To identify studies pertinent to this meta-analysis, we conducted a comprehensive search of PubMed, Embase, Web of Science, Wanfang, and China National Knowledge Infrastructure (CNKI) databases using an extensive array of search terms, which included: (1) “chemerin”; and (2) “inflammatory bowel disease” OR “IBD” OR “ulcerative colitis” OR “Crohn disease” OR “Crohn’s disease”. The search was restricted to studies involving human subjects and included only full-length articles published in English or Chinese in peer-reviewed journals. Additionally, a manual screening of the references of relevant original and review articles was performed to identify any additional eligible studies. The literature search covered the period from the inception of the databases up to February 10, 2024.

### Inclusion and exclusion criteria

The inclusion criteria for potential studies were defined according to the PICOS framework:

P (Population): Patients diagnosed with IBD, including CD or UC, confirmed by clinical, endoscopic, histological, or radiological criteria. Studies must include participants without comorbidities known to influence serum chemerin levels, such as type 2 diabetes, metabolic syndrome, cardiovascular diseases, chronic kidney disease, or other autoimmune/inflammatory conditions.

I (Intervention/Exposure): Measurement of blood chemerin levels in patients with IBD.

C (Comparison): For the primary outcome, healthy individuals without IBD or other inflammatory/metabolic diseases. For the secondary outcome, IBD patients in the active phase of the disease compared to those in the remission (non-active) phase.

O (Outcomes): The primary outcome is the difference in serum chemerin levels between IBD patients and healthy controls. The secondary outcome is the difference in serum chemerin levels between patients with active IBD and those in remission.

S (Study Design): Observational studies, including cross-sectional, case-control, and cohort studies, that provide original data.

Studies were excluded if they were reviews, editorials, meta-analyses, preclinical research, not including patients with IBD, lack of controls, or did not report blood levels of chemerin. In cases of overlapping populations, the study with the largest sample size was included in the meta-analysis.

### Study quality assessment and data extraction

The literature search, study selection, quality assessment, and data extraction were independently and in duplicate performed by two authors, with any discrepancies resolved through discussion with the corresponding author. Study quality was assessed using the Newcastle–Ottawa Scale (NOS) [36], which evaluates the selection of study participants, control of confounding factors, and outcome measurement and analysis, with scores ranging from 1 to 9, with a score of 9 indicating the highest quality. Data extracted for analysis included study characteristics (author, year, country, and design), participant details (numbers of patients with active/non-active IBD, numbers of healthy controls, and the mean age, sex, and body mass index [BMI] of the included participants), measuring methods for blood chemerin levels, and variables matched between cases and controls. Where available, information regarding the specific kit used for chemerin measurement (manufacturer and catalog or lot number) and the corresponding measurement units was also extracted from each study.

### Statistical analyses

The primary outcome of this study was to compare the blood level of chemerin between patients with IBD and healthy controls, and the secondary outcome was to compare the blood level of chemerin between patients with active and non-active IBD. The standardized mean difference (SMD) with 95% confidence intervals (CI) was used to represent the difference in chemerin levels, as although all included studies measured serum chemerin using enzyme-linked immunosorbent assay (ELISA), the assay kits were not entirely consistent across studies, and the reported units of measurement varied [35]. To assess for heterogeneity, we used the Cochrane Q test and I² statistics [37], with I² < 25%, 25 ~ 75%, and > 75% indicating mild, moderate, and substantial heterogeneity. A random-effects model was applied to synthesize the results, accounting for study variability [35]. A sensitivity analysis was conducted by sequentially excluding individual studies to evaluate the robustness of the findings. Predefined subgroup analyses were conducted to assess the consistency of results across studies involving CD and UC, studies with different NOS scores, studies conducted in Asian versus non-Asian countries, and studies with versus without BMI matching between cases and controls. Publication bias was evaluated using funnel plots and visual inspection for asymmetry, supplemented by Egger’s regression test [38]. Meta-analyses, subgroup analyses, and sensitivity analyses were performed using RevMan (version 5.1; Cochrane Collaboration, Oxford, UK) and Egger’s regression test for publication bias was performed using Stata software (version 12.0; Stata Corporation, College Station, TX, USA). In addition, we assessed the certainty of evidence for the two primary outcomes using the GRADE (Grading of Recommendations Assessment, Development and Evaluation) approach, which considers factors such as risk of bias, inconsistency, indirectness, imprecision, and publication bias [39]. Given the observational design of the included studies, the initial certainty was rated as low, with potential upgrades based on study quality and consistency.

## Results

### Study identification

**Fig. 1:**
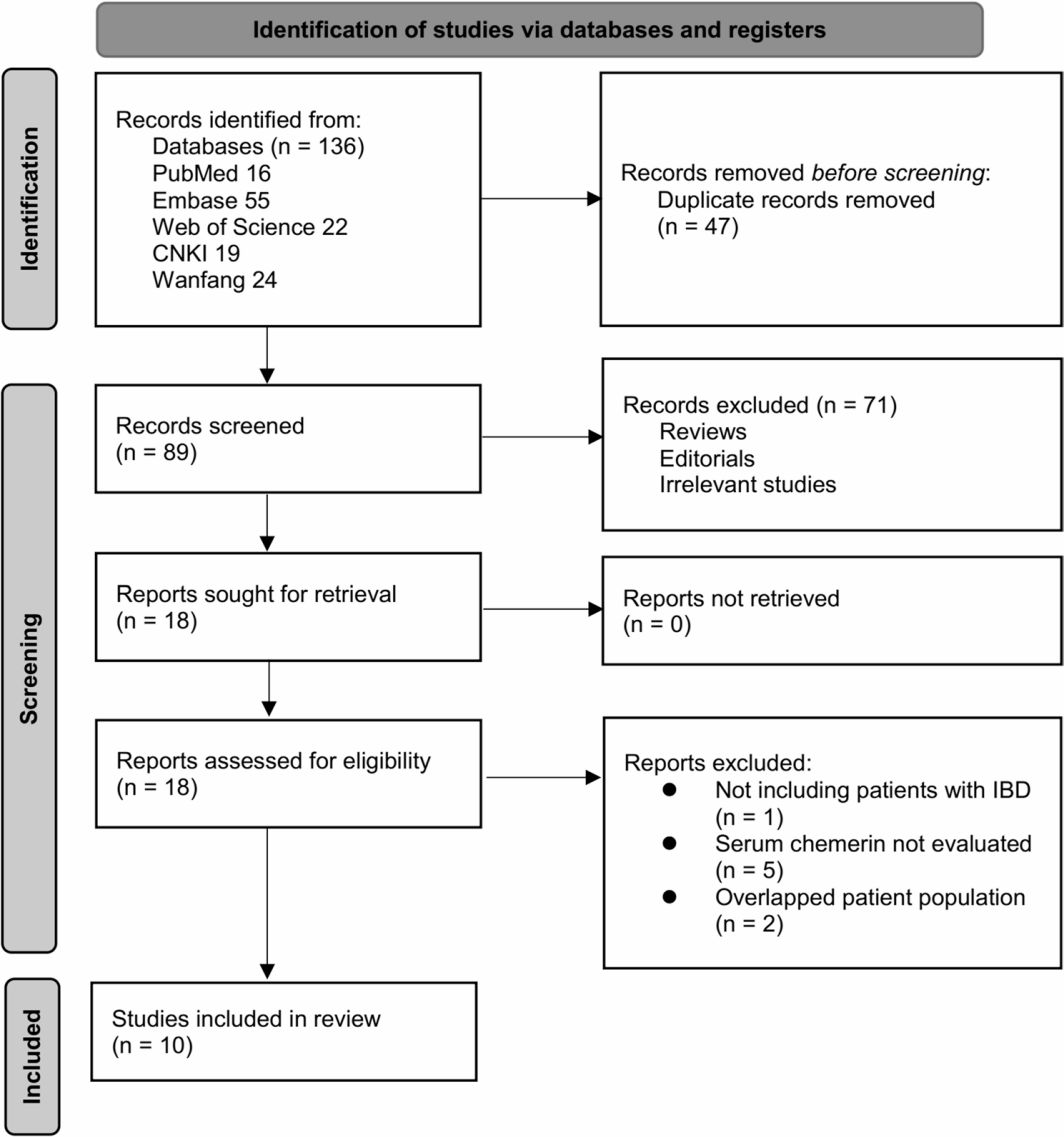
Flowchart of database search and study inclusion

The study selection process is summarized in Fig. 1. Initially, a total of 136 potentially relevant records were identified from the three databases searched and citations of related articles, after removing 47 duplicates. A screening of titles and abstracts led to the exclusion of 71 articles that did not align with the objectives of the meta-analysis. The full texts of the remaining 18 articles were independently reviewed by two authors, resulting in the exclusion of eight studies for various reasons, as detailed in Fig. 1. Ultimately, ten studies were included in the quantitative analysis [21–30].

### Overview of the study characteristics

Table 1 presents the summarized characteristics of the studies included in the meta-analysis. Because eight studies [21, 23, 24, 26–30] reported comparisons of patients with CD and UC versus healthy controls separately, these data were independently included in the meta-analysis. To avoid double-counting the control group, the sample size of the healthy controls was proportionally divided, as recommended by the Cochrane Handbook [35]. A total of ten case-control studies with 18 datasets were included in the meta-analysis [21–30]. These studies were published between 2010 and 2024, and performed in Germany, China, Poland, and Greece. Overall, this meta-analysis included 799 patients with CD, 520 patients with UC, and 609 patients with healthy controls. Among the included patients with IBD, 756 had active disease, while 563 were in the remission phase. The mean ages of the included participants were 23.8 to 50.2 years, with the proportions of men varying from 24.5 to 73.4%. The blood levels of chemerin were measured via ELISA in all the included studies. Age and sex were matched between cases and controls in all the included studies, and BMI was also matched in five studies [21, 22, 27, 29, 30]. The included studies achieved NOS scores ranging from seven to nine, reflecting a generally high quality of methodology and reporting (Table 2).

**Table 1:** Characteristics of the included studies

Study	Country	Study design	No. of patients with CD	No. of patients with UC	No. of patients with active IBD	No. of patients with non-active IBD	No. of Healthy control	Mean age (years)	Men (%)	BMI (kg/m2)	Methods, kit and unit for measuring serum chemerin	Variables matched or adjusted
Weigert 2010 CD [20]	Germany	CC	230	0	148	82	40	41	73.4	22.9	ELISA, R&D, ng/mL	Age, sex, and BMI
Weigert 2010 UC [20]	Germany	CC	0	80	31	49	40	41.7	47.5	23	ELISA, R&D, ng/mL	Age, sex, and BMI
Gu 2010 CD [27]	China	CC	50	0	50	0	25	34.8	45.3	NR	ELISA, manufacturer NR, µg/L	Age and sex
Gu 2010 UC [27]	China	CC	0	50	50	0	25	35.9	44	NR	ELISA, manufacturer NR, µg/L	Age and sex
Gong 2011 CD [21]	China	CC	45	0	45	0	45	32.9	48.9	24	ELISA, manufacturer NR, µg/L	Age, sex, and BMI
Waluga 2014 CD [28]	Poland	CC	24	0	24	0	8	30.8	46.9	21.9	ELISA, BioVendor, ng/mL	Age, sex, BMI, and WHR
Waluga 2014 UC [28]	Poland	CC	0	16	16	0	8	32.1	45.8	22.9	ELISA, BioVendor, ng/mL	Age, sex, BMI, and WHR
Terzoudis 2016 CD [22]	Greece	CC	68	0	13	55	49	44.6	51.5	NR	ELISA, manufacturer NR, ng/mL	Age and sex
Terzoudis 2016 UC [22]	Greece	CC	0	52	17	35	49	50.2	55.8	NR	ELISA, manufacturer NR, ng/mL	Age and sex
Xu 2017 CD [23]	China	CC	72	0	52	20	34	34	38.9	NR	ELISA, R&D, ng/mL	Age and sex
Xu 2017 UC [23]	China	CC	0	49	37	12	34	39	24.5	NR	ELISA, R&D, ng/mL	Age and sex
Sochal 2021 CD [29]	Poland	CC	45	0	29	16	21	35	50	23.5	ELISA, Finetest, ng/mL	Age, sex, and BMI
Sochal 2021 UC [29]	Poland	CC	0	32	19	13	21	35	50	23.5	ELISA, Finetest, ng/mL	Age, sex, and BMI
Lu 2021 [24]	China	CC	88	54	80	62	140	46	62.8	NR	ELISA, BioTNT, ng/mL	Age and sex
Yang 2022 CD [25]	China	CC	85	0	28	57	35	23.8	53.3	NR	ELISA, manufacturer NR, ng/mL	Age and sex
Yang 2022 UC [25]	China	CC	0	65	22	43	35	31.2	54	NR	ELISA, manufacturer NR, ng/mL	Age and sex
Li 2024 CD [26]	China	CC	92	0	41	51	0	50.2	65.2	20.9	ELISA, manufacturer Jingmei Biotech, ng/mL	Age, sex, and BMI
Li 2024 UC [26]	China	CC	0	122	54	68	0	52	47.5	21.5	ELISA, manufacturer Jingmei Biotech, ng/mL	Age, sex, and BMI

For studies reporting separate data for Crohn’s disease (CD) and ulcerative colitis (UC), each subgroup is presented as an individual row (e.g., “Weigert 2010 CD” and “Weigert 2010 UC”) for consistency with subsequent meta-analysis results. These datasets were independently included in the meta-analysis, as described in the Methods part

*CD* Crohn’s disease, *UC* Ulcerative colitis, *CC* Case-control studies, *IBD* Inflammatory bowel disease, *BMI* Body mass index, *NR* Not reported, *ELISA* Enzyme-linked immunosorbent assay

**Table 2:** Study quality evaluation via the Newcastle-Ottawa scale

Studies	Adequate definition of the cases	Representativeness of the cases	Selection of Controls	Definition of Controls	Controlled for age and sex	Controlled for other confoundings	Ascertainment of the exposure	Same method of ascertainment of exposure for cases and controls	Non-response rate	Overall
Weigert 2010 CD [20]	1	0	1	1	1	1	1	1	1	8
Weigert 2010 UC [20]	1	0	1	1	1	1	1	1	1	8
Gu 2010 CD [27]	1	0	1	1	1	0	1	1	1	7
Gu 2010 UC [27]	1	0	1	1	1	0	1	1	1	7
Gong 2011 CD [21]	1	0	1	1	1	1	1	1	1	8
Waluga 2014 CD [28]	1	0	1	1	1	1	1	1	1	8
Waluga 2014 UC [28]	1	0	1	1	1	1	1	1	1	8
Terzoudis 2016 CD [22]	1	0	1	1	1	0	1	1	1	7
Terzoudis 2016 UC [22]	1	0	1	1	1	0	1	1	1	7
Xu 2017 CD [23]	1	0	1	1	1	0	1	1	1	7
Xu 2017 UC [23]	1	0	1	1	1	0	1	1	1	7
Sochal 2021 CD [29]	1	0	1	1	1	1	1	1	1	8
Sochal 2021 UC [29]	1	0	1	1	1	1	1	1	1	8
Lu 2021 [24]	1	1	1	1	1	0	1	1	1	8
Yang 2022 CD [25]	1	0	1	1	1	0	1	1	1	7
Yang 2022 UC [25]	1	0	1	1	1	0	1	1	1	7
Li 2024 CD [26]	1	1	1	1	1	1	1	1	1	9
Li 2024 UC [26]	1	1	1	1	1	1	1	1	1	9

*CD* Crohn’s disease, *UC* Ulcerative colitis

### Difference of blood chemerin between IBD patients and controls

**Fig. 2:**
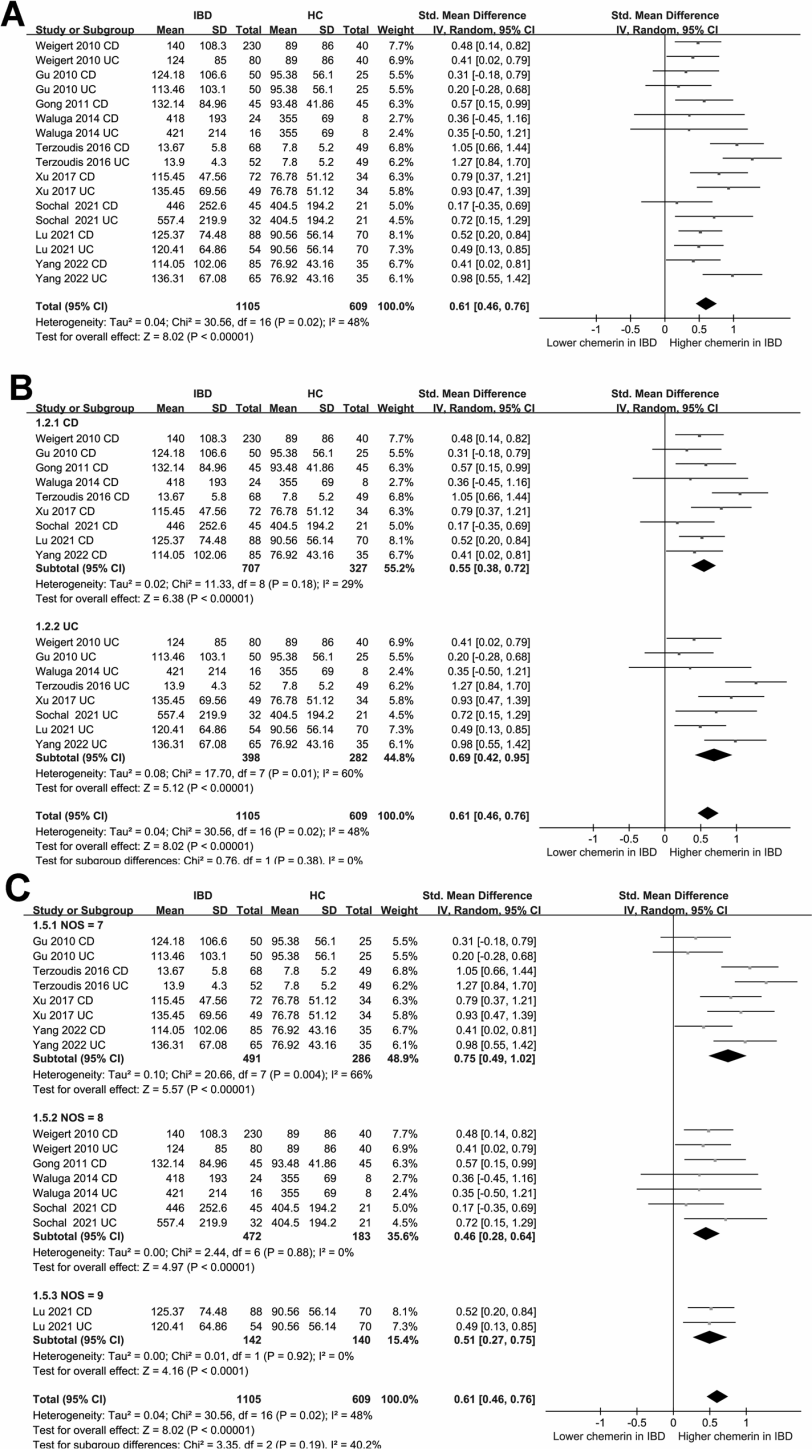
Forest plots for the meta-analysis comparing the blood level of chemerin between IBD patients and healthy controls. **A** overall meta-analysis; **B** subgroup analysis according to type of IBD; **C** subgroup analysis according to NOS scores

**Fig. 3:**
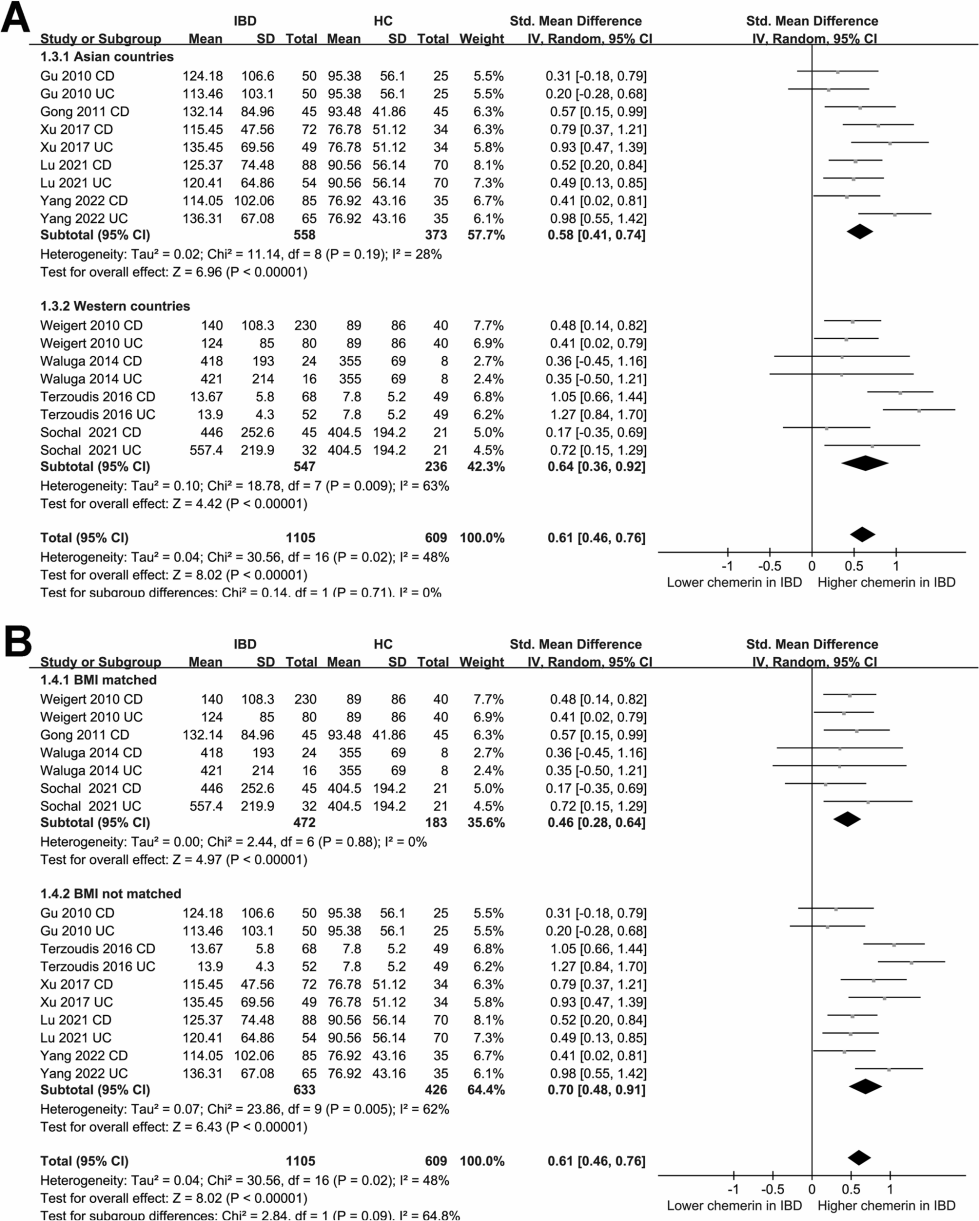
Forest plots for the subgroup analysis comparing the blood level of chemerin between IBD patients and healthy controls. **A** subgroup analysis according to study country; **B** subgroup analysis according to whether BMI was matched

The meta-analysis involving nine studies [21–26, 28–30] of 17 datasets showed that overall, patients with IBD had a higher blood level of chemerin as compared to healthy controls (SMD: 0.46, 95% CI: 0.46 to 0.76, *p* < 0.001; Fig. 2A) with moderate heterogeneity (*p* for Cochrane Q test = 0.02; I^2^ = 48%). Further sensitivity analyses, conducted by omitting one dataset at a time, yielded similar results (SMD: 0.57 to 0.63, *p* all < 0.001; Table 3). Subsequent subgroup analyses yielded similar results among patients with CD and UC (*p* for subgroup difference = 0.38; Fig. 2B), in studies with different NOS scores (*p* for subgroup difference = 0.19; Fig. 2C), in both Asian and non-Asian studies (*p* for subgroup difference = 0.71; Fig. 3A), and in studies with and without BMI matching (*p* for subgroup difference = 0.09; Fig. 3B).

**Table 3:** Results of sensitivity analyses

Dataset excluded	Difference of blood chemerin level between IBD patients and healthy controls			
	SMD (95% CI)	*p* values for effect	*p* values for heterogeneity	I^2^
Weigert 2010 CD [20]	0.62 [0.46, 0.78]	< 0.001	0.01	50%
Weigert 2010 UC [20]	0.62 [0.47, 0.78]	< 0.001	0.01	49%
Gu 2010 CD [27]	0.63 [0.47, 0.78]	< 0.001	0.02	48%
Gu 2010 UC [27]	0.63 [0.48, 0.78]	< 0.001	0.02	46%
Gong 2011 CD [21]	0.61 [0.45, 0.77]	< 0.001	0.01	51%
Waluga 2014 CD [28]	0.62 [0.46, 0.77]	< 0.001	0.01	50%
Waluga 2014 UC [28]	0.61 [0.46, 0.77]	< 0.001	0.01	50%
Terzoudis 2016 CD [22]	0.58 [0.43, 0.72]	< 0.001	0.05	41%
Terzoudis 2016 UC [22]	0.57 [0.44, 0.70]	< 0.001	0.14	28%
Xu 2017 CD [23]	0.60 [0.44, 0.75]	< 0.001	0.01	50%
Xu 2017 UC [23]	0.59 [0.44, 0.74]	< 0.001	0.02	48%
Sochal 2021 CD [29]	0.63 [0.48, 0.78]	< 0.001	0.02	46%
Sochal 2021 UC [29]	0.60 [0.45, 0.76]	< 0.001	0.01	51%
Lu 2021 CD [24]	0.62 [0.45, 0.78]	< 0.001	0.01	50%
Lu 2021 CD [24]	0.62 [0.46, 0.78]	< 0.001	0.01	50%
Yang 2022 CD [25]	0.62 [0.47, 0.78]	< 0.001	0.01	49%
Yang 2022 UC [25]	0.58 [0.43, 0.73]	< 0.001	0.02	46%
Dataset excluded	Difference of blood chemerin level between active and non-active IBD patients			
	SMD (95% CI)	*p* values for effect	*p* values for heterogeneity	I^2^
Weigert 2010 CD [20]	0.41 [0.25, 0.57]	< 0.001	0.40	5%
Weigert 2010 UC [20]	0.37 [0.17, 0.57]	< 0.001	0.07	44%
Xu 2017 CD [23]	0.34 [0.15, 0.54]	< 0.001	0.08	41%
Xu 2017 UC [23]	0.31 [0.15, 0.48]	< 0.001	0.23	23%
Sochal 2021 CD [29]	0.31 [0.14, 0.48]	< 0.001	0.21	25%
Sochal 2021 UC [29]	0.35 [0.16, 0.55]	< 0.001	0.07	43%
Lu 2021 [24]	0.36 [0.15, 0.57]	< 0.001	0.07	43%
Yang 2022 CD [25]	0.38 [0.18, 0.59]	< 0.001	0.07	43%
Yang 2022 UC [25]	0.38 [0.18, 0.58]	< 0.001	0.07	43%
Li 2024 CD [26]	0.38 [0.17, 0.58]	< 0.001	0.07	44%
Li 2024 UC [26]	0.37 [0.16, 0.58]	< 0.001	0.06	44%

*SMD* Standardized mean difference, *CI* Confidence interval, *CD* Crohn’s disease, *UC* Ulcerative colitis, *IBD* Inflammatory bowel disease

### Difference of blood chemerin between active and non-active IBD patients

**Fig. 4:**
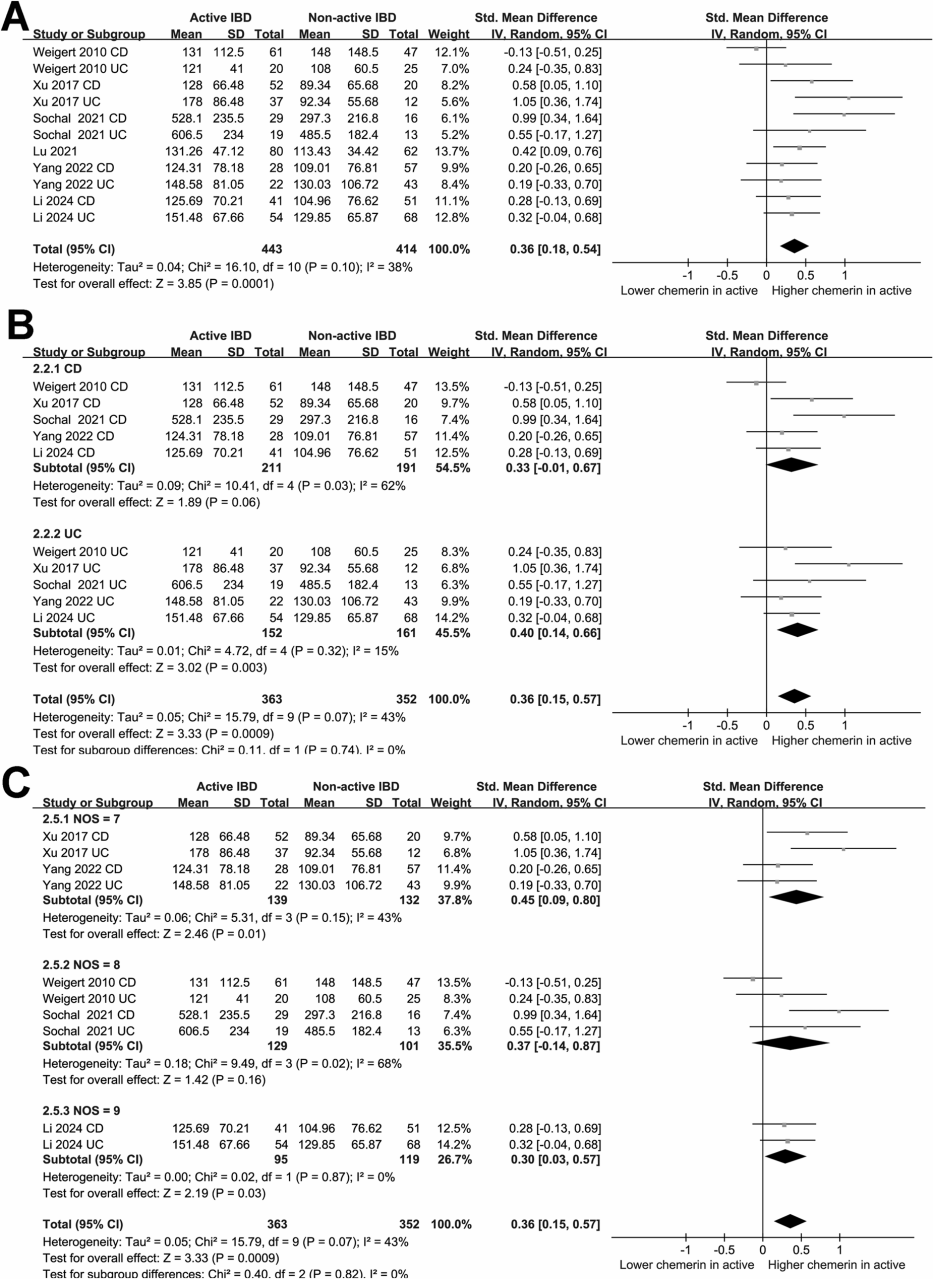
Forest plots for the meta-analysis comparing the blood level of chemerin between active and non-active IBD patients. **A** overall meta-analysis; **B** subgroup analysis according to type of IBD; **C** subgroup analysis according to NOS scores

**Fig. 5:**
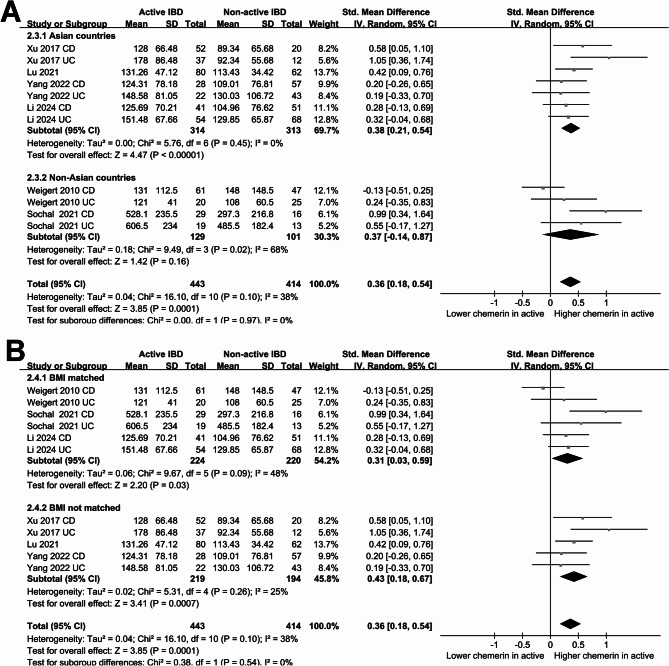
Forest plots for the subgroup analysis comparing the blood level of chemerin between active and non-active IBD patients. **A** subgroup analysis according to study country; **B** subgroup analysis according to whether BMI was matched

The pooled results including six studies of 11 datasets suggested that patients with active IBD were associated with a higher blood level of chemerin compared to patients with non-active IBD (SMD: 0.36, 95% CI: 0.15 to 0.57, *p* < 0.001; Fig. 4A) with moderate heterogeneity (*p* for Cochrane Q test = 0.10; I^2^ = 38%). Further sensitivity analyses, conducted by excluding one dataset at a time, yielded similar results (SMD: 0.31 to 0.41, *p* all < 0.001; Table 3). In addition, subgroup analyses retrieve consistent results in patients with CD and UC (*p* for subgroup difference = 0.74; Fig. 4B), in studies with different NOS scores (*p* for subgroup difference = 0.82; Fig. 4C), in both Asian and non-Asian studies (*p* for subgroup difference = 0.97; Fig. 5A), and in studies with and without BMI matching (*p* for subgroup difference = 0.54; Fig. 5B).

### Publication bias

**Fig. 6:**
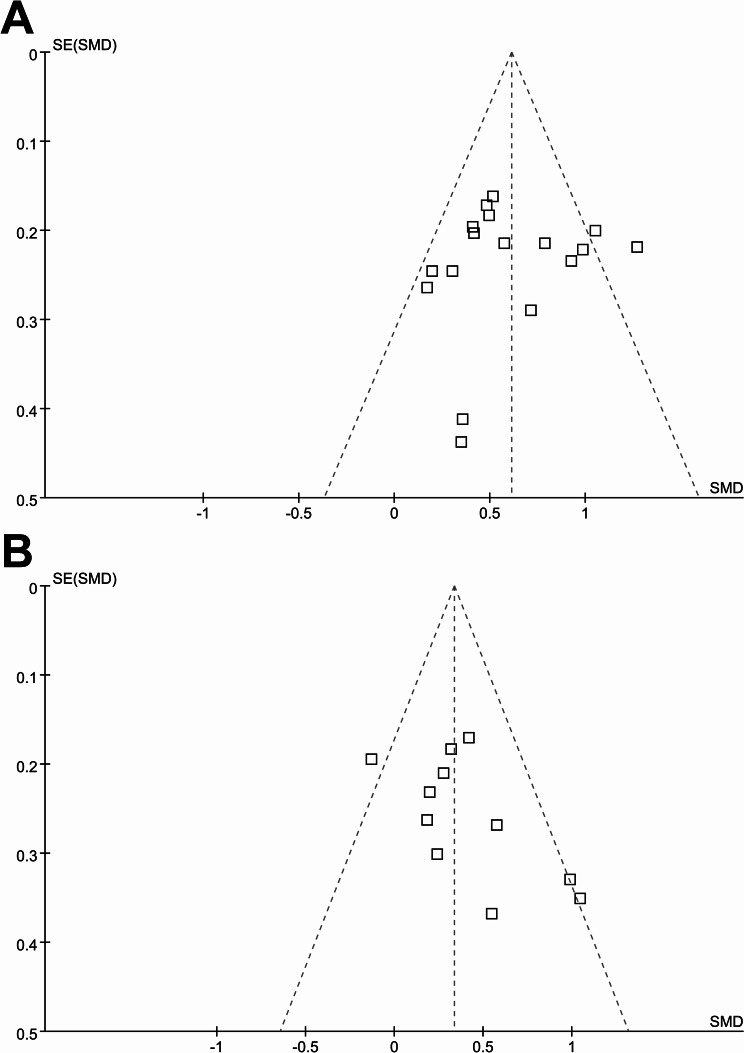
Funnel plots for estimating the potential publication biases underlying the meta-analyses. **A** funnel plots for the meta-analysis comparing the blood level of chemerin between IBD patients and healthy controls; **B** funnel plots for the meta-analysis comparing the blood level of chemerin between active and non-active IBD patients

The funnel plots for the meta-analysis assessing the differences of blood chemerin between IBD patients and healthy controls, as well as between patients with active and non-active IBD are shown in Fig. 6A and B. Visual inspection of the plots revealed symmetry, indicating a low risk of publication bias, which was further corroborated by Egger’s regression analysis (*p* = 0.27 and 0.39, respectively).

### Certainty of evidence

According to the GRADE assessment, the certainty of evidence for both main outcomes—elevated chemerin levels in IBD patients versus healthy controls, and in active versus non-active IBD—was rated as moderate. This rating reflects the generally high quality and consistency of the included observational studies, absence of serious risk of bias, and lack of publication bias. Details are presented in Supplemental File 2.

## Discussion

This meta-analysis provides comprehensive evidence supporting an association between elevated blood chemerin levels and IBD, including both CD and UC. Our pooled analysis of 18 datasets from 10 case-control studies demonstrated that patients with IBD had significantly higher circulating chemerin levels compared to healthy individuals. Additionally, chemerin levels were significantly elevated in patients with active disease compared to those in remission. These associations remained robust across multiple sensitivity analyses and subgroup comparisons, highlighting the potential of chemerin as a promising biomarker for the presence and activity of IBD.

The observed elevation of chemerin in IBD patients and particularly in those with active disease may be explained by its role as a chemoattractant and regulator of immune cell trafficking. Chemerin is known to mediate the recruitment of dendritic cells and macrophages to sites of inflammation through CMKLR1 signaling [40]. These immune cells play central roles in the pathogenesis of IBD by producing pro-inflammatory cytokines such as TNF-α, IL-6, and IL-1β, which contribute to tissue injury and chronic inflammation [41, 42]. Furthermore, chemerin has been shown to regulate the balance between pro-inflammatory and anti-inflammatory responses, influence adipokine secretion, and potentially interact with the gut microbiome, all of which are implicated in IBD pathophysiology [19, 43]. Its production by adipocytes and epithelial cells in response to inflammatory stimuli may suggest that chemerin may act as both a marker and mediator of intestinal inflammation [44].

Subgroup analyses revealed consistent results regardless of IBD subtype (CD or UC), study quality scores, geographic location (Asian vs. non-Asian), or BMI matching between cases and controls. Notably, subgroup analyses by region and BMI matching showed consistent results, suggesting that geographic variability or differences in adiposity could account for inter-study heterogeneity. Chemerin is secreted by adipose tissue and is modulated by metabolic factors; thus, studies that did not match for BMI might overestimate the IBD–chemerin association [45]. Obesity has been independently linked with altered immune responses and low-grade systemic inflammation, potentially confounding the relationship between chemerin and IBD [46]. Similarly, regional differences could reflect distinct genetic or environmental influences on systemic inflammation. However, the absence of significant subgroup differences suggests that elevated chemerin in IBD patients is a consistent finding across diverse study settings and populations, reinforcing its potential relevance as a broadly applicable biomarker. Nonetheless, the influence of metabolic status on chemerin levels in IBD warrants further exploration in future studies with individual-level data.

This meta-analysis has several notable strengths. We conducted an up-to-date and comprehensive literature search across multiple international and Chinese databases, allowing for broad inclusion and reduced language bias. All included studies were observational in design but matched participants for age and sex, reducing potential confounding by demographic factors. We applied rigorous methodological standards for study selection, quality assessment, and statistical synthesis, and performed multiple sensitivity and predefined subgroup analyses to test the robustness of our findings. These efforts enhance the validity and reliability of our conclusions. Importantly, most of the included studies were of moderate to high quality, as reflected by NOS scores ranging from 7 to 9, with over half scoring 8 or higher. This enhances confidence in the methodological rigor of the pooled results. Furthermore, subgroup analysis by study quality did not reveal significant differences, reinforcing the robustness of our findings. To further evaluate the robustness of our findings, we conducted a GRADE assessment, which rated the certainty of evidence as moderate for both primary outcomes. Although observational studies typically begin at a low level of certainty, the absence of serious methodological limitations, moderate to high NOS scores, consistent findings across studies, and lack of publication bias justified an upgrade. This further supports the reliability of our conclusions.

However, several limitations should be acknowledged. First, all included studies were observational in nature, limiting our ability to infer a causal relationship between chemerin levels and IBD. Although associations were observed, residual confounding from unmeasured or inadequately adjusted variables (e.g., dietary habits, medication use, disease duration, smoking status) may exist. Second, analyses were based on study-level data, which restricted our capacity to explore how patient-level characteristics (e.g., age, sex, disease duration, comorbidities) might modify the observed associations. Third, although the included studies used ELISA to measure chemerin, there may have been variability in assay kits, calibration standards, and sample handling procedures, contributing to inter-study heterogeneity. Additionally, although chemerin levels are known to be influenced by metabolic and inflammatory conditions such as obesity, smoking, and medication use, these variables were inconsistently reported across studies and could not be fully accounted for in our analysis [47]. The observed associations may thus be partially confounded by these unmeasured factors. For example, corticosteroids and biologics may independently modulate chemerin levels, and active smokers may exhibit altered adipokine profiles irrespective of IBD status [48]. These limitations underscore the need for well-controlled prospective studies to clarify the independent role of chemerin in IBD. Finally, due to limited reporting, we were unable to analyze the influence of treatment regimens, disease severity scores, or gut microbiota profiles on circulating chemerin levels. Despite using SMDs to standardize chemerin levels across studies with different measurement scales, moderate heterogeneity persisted. This may be attributed to differences in ELISA assay kits, sample handling protocols, population characteristics (e.g., age, disease duration, comorbidities), or variability in defining disease activity and remission. Although subgroup and sensitivity analyses reduced concerns about major bias, these methodological and clinical differences likely contributed to the observed heterogeneity. Individual-patient data based meta-analyses may be considered in the future to address the source of heterogeneity. Finally, we did not perform meta-regression due to the limited number of included studies, lack of uniformly reported continuous variables, and overlap between categorical covariates and prespecified subgroup analyses. These constraints limited the feasibility and added value of conducting meta-regression in this context.

From a clinical perspective, our findings suggest that chemerin has potential utility as a non-invasive biomarker for IBD diagnosis and monitoring. Its elevation in both the presence and activity of IBD highlights its possible value in distinguishing active from inactive disease, which could support therapeutic decision-making and disease surveillance. Nevertheless, given the early stage of evidence, chemerin should not yet be considered a standalone diagnostic or monitoring tool. Further prospective studies are needed to clarify its temporal relationship with disease onset and flare-ups, and to evaluate its performance in conjunction with established inflammatory markers such as C-reactive protein, fecal calprotectin, and endoscopic indices [49]. Future research should aim to validate our findings in larger, well-characterized cohorts with longitudinal follow-up and detailed clinical data. The use of individual patient data meta-analyses could allow for more nuanced assessments of effect modifiers and confounders. Moreover, mechanistic studies investigating the specific pathways through which chemerin contributes to mucosal inflammation, immune cell activation, and gut barrier dysfunction may help elucidate its role in IBD pathogenesis. Exploring whether chemerin levels respond to treatment or predict therapeutic response—particularly with biologics or nutritional therapy—would also provide important clinical insights.

## Conclusions

In conclusion, this meta-analysis demonstrates that blood chemerin levels are significantly elevated in patients with IBD and are further increased during active disease. These findings support the potential role of chemerin as a biomarker reflecting the presence and activity of IBD. While promising, the current evidence remains preliminary, and further high-quality studies are needed to clarify the clinical value and underlying mechanisms of chemerin in IBD.

## Supplementary Information

Supplementary Material 1.

Supplementary Material 2.

## Data Availability

The data underlying this article are available in the article.
