# Supplementary material for: Chemerin as a biomarker of inflammatory bowel diseases: a meta-analysis
Source: BMC Gastroenterol. 2025 Sep 26;25:662. doi: 10.1186/s12876-025-04132-2 (PMC12465509; doi:10.1186/s12876-025-04132-2)
Supplement: Supplementary file 1 — Supplementary Material 1. [file 12876_2025_4132_MOESM1_ESM.docx]

PubMed

("chemerin"[Title/Abstract] OR "RARRES2"[Title/Abstract] OR "tazarotene-induced gene 2 protein"[Title/Abstract])

AND

("Inflammatory Bowel Diseases"[MeSH] OR "Ulcerative Colitis"[MeSH] OR "Crohn Disease"[MeSH]

OR "inflammatory bowel disease"[Title/Abstract] OR "IBD"[Title/Abstract]

OR "ulcerative colitis"[Title/Abstract] OR "Crohn disease"[Title/Abstract] OR "Crohn's disease"[Title/Abstract])

Embase

('chemerin'/exp OR chemerin:ti,ab OR 'RARRES2':ti,ab OR 'tazarotene induced gene 2 protein':ti,ab)

AND

('inflammatory bowel disease'/exp OR 'ulcerative colitis'/exp OR 'Crohn disease'/exp

OR 'inflammatory bowel disease':ti,ab OR IBD:ti,ab

OR 'ulcerative colitis':ti,ab OR 'Crohn disease':ti,ab OR 'Crohn's disease':ti,ab)

Web of Science

TS=("chemerin" OR "RARRES2" OR "tazarotene-induced gene 2 protein")

AND

TS=("inflammatory bowel disease" OR "IBD" OR "ulcerative colitis" OR "Crohn disease" OR "Crohn's disease")

Wanfang

全部:(chemerin OR 趋化素) AND 全部:(炎性肠病 OR 克罗恩病 OR 溃疡性结肠炎)

CNKI

(chemerin OR 趋化素) AND (炎性肠病 OR 克罗恩病 OR 溃疡性结肠炎)
