# Supplementary material for: Chemerin as a biomarker of inflammatory bowel diseases: a meta-analysis
Source: BMC Gastroenterol. 2025 Sep 26;25:662. doi: 10.1186/s12876-025-04132-2 (PMC12465509; doi:10.1186/s12876-025-04132-2)
Supplement: Supplementary file 2 — Supplementary Material 2. [file 12876_2025_4132_MOESM2_ESM.docx]

Supplemental File 2 Summarized certainty of evidence using the GRADE system

| Outcome | No. of studies | Study design | Risk of bias | Inconsistency | Indirectness | Imprecision | Publication bias | Overall certainty |
| --- | --- | --- | --- | --- | --- | --- | --- | --- |
| Higher blood chemerin in IBD vs. healthy controls | 9 studies (17 datasets) | Case-control | Not serious | Not serious | Not serious | Not serious | None detected | Moderate |
| Higher blood chemerin in active vs. non-active IBD | 6 studies (11 datasets) | Case-control | Not serious | Not serious | Not serious | Not serious | None detected | Moderate |

GRADE, Grading of Recommendations, Assessment, Development and Evaluation; IBD, inflammatory bowel disease;

Specific reasons for each GRADE domain, including:

Risk of bias: Downgraded if a significant proportion of studies had unclear or high risk of bias in key domains (e.g., random sequence generation, allocation concealment, or selective reporting).

Inconsistency: Downgraded if substantial heterogeneity was observed (I² > 50%) and could not be explained by subgroup analyses or meta-regression.

Indirectness: Evaluated but not downgraded, as all included studies directly assessed the population and outcomes of interest.

Imprecision: Downgraded if confidence intervals were wide, overlapping no effect, or if the overall sample size was small.

Publication bias: Assessed using funnel plots and Egger’s test; downgraded if significant asymmetry suggested potential bias.
